# Curvilinear association between waist-to-height ratio and cardiorespiratory fitness: a cross-sectional study based on nationwide data from Chinese children and adolescents

**DOI:** 10.1186/s13102-024-00868-8

**Published:** 2024-03-28

**Authors:** Jiehao Dong, Pengwei Song, Zhen Zhang, Jia Zhang

**Affiliations:** 1 School of Physical Education and Health, Guangxi Normal University, 541006 Guilin, Guangxi China; 2 School of Physical Education, Guangxi Science and Technology Normal University, 546199 Laibin, Guangxi China; 3 School of Physical Education, Chongqing University, 400044 Chongqing, China

**Keywords:** Waist-to-height ratio, Children and adolescents, Cardiorespiratory Fitness, Associations, Cross-sectional analyses

## Abstract

**Background:**

Waist-to-height ratio (WHtR) is considered an important summary indicator for assessing the health of children and adolescents. However, there are fewer studies addressing the association between WHtR and cardiorespiratory fitness (CRF). Deriving an optimal WHtR would play an important role in promoting CRF in children and adolescents. Our aim was to analyze the association between WHtR and CRF and determine the optimal value of WHtR.

**Methods:**

In this study, 37,081 (19,125 boys, 51.6%) children and adolescents aged 7–18 years in five regions of China were tested for WHtR and 20-m shuttle run test (20 m SRT). One-way ANOVA was used to compare maximum oxygen uptake (VO_2max_) among children and adolescents with different WHtRs and effect sizes were used to analyze differences between groups. Curvilinear regression was used to analyse the curvilinear relationship that exists between WHtR and VO_2max_.

**Results:**

In Chinese children and adolescents, the WHtR of boys was higher than that of girls by 0.01, and the difference was statistically significant (*P* < 0.001). Overall, in the age groups of 7–9, 10–12, 13–15, and 16–18 years old, the differences in VO_2max_ comparisons between different WHtR groups were statistically significant (*P* < 0.001). In Chinese children and adolescents in the age groups of 7–9, 10–12, 13–15, and 16–18 years old, VO_2max_ levels were highest when the WHtR was 0.34, 0.32, 0.39, and 0.41, respectively.

**Conclusions:**

There is a curvilinear association between WHtR and CRF in Chinese children and adolescents. Both lower and higher WHtR led to a decrease in VO_2max_ in children and adolescents.

## Introduction

Cardio-respiratory Fitness (CRF) is the body’s ability to provide oxygen to the muscles during sustained exercise, and is a comprehensive reflection of the body’s aerobic capacity of the heart, lung, blood vessels and tissue cells [1]. Maximum oxygen uptake (VO_2max_) is a direct index commonly used internationally to assess to CRF, so VO_2max_ was used in this study to reflect CRF in children and adolescents. VO_2max_ can be obtained from subjects by various testing methods such as running table test, 20-m shuttle run test (20-mSRT), 12 min run test, etc [2, 3]... The results of several epidemiologic investigations have shown that declines in CRF in children and adolescents are strongly associated with cardiovascular health, increased risk of metabolic diseases, decreased academic performance, and lower levels of executive functioning [4–6]. Given the importance of CRF to the health of children and adolescents, the American Heart Association has identified CRF as the 5th most important clinical life characteristic of the human body, thus demonstrating the importance of its CRF to life and health [7]. However, studies have confirmed that CRF levels in children and adolescents have shown a significant decline worldwide in recent decades. In developed countries in Europe and the United States, CRF levels in children and adolescents have decreased by nearly 15%, and in some emerging developed countries, such as South Korea and Singapore, CRF levels have decreased by nearly 30% [8]. A study shows that CRF levels in British schoolchildren are declining at a rate of 0.95% per year, twice the world average of 0.43% [9]. An analysis of longitudinal trends among 228 children and adolescents aged 7 to 17 years in New York City public schools found that unhealthy levels of cardiorespiratory fitness among U.S. children and adolescents increased from 15.1 to 23.6% over the 10-year period 2006–2016 [10]. The cardiorespiratory fitness level of Chinese children and adolescents is also not optimistic. The results of the 2019 National Survey on Students’ Physical Fitness and Health show that although the indicators of muscle strength and CRF of Chinese students have improved, they are still at a low level, and the improvement of CRF should be highly emphasized [11]. Overall, it can be seen that there is a general downward trend in CRF among children and adolescents around the world. Therefore, an effective understanding of the influencing factors affecting CRF is an important tool to effectively improve CRF. Previous studies have confirmed that CRF is influenced by the combination of multiple indicators such as physical activity, sedentary lifestyle, screen time, diet, sleep, waist circumference, BMI in children and adolescents [12–14]. However, fewer previous studies have addressed the relationship between WHtR and VO_2max_ metrics reflecting CRF in children and adolescents.

In evaluating childhood and adolescent obesity, body mass index (BMI) has been adopted by international organizations such as the World Health Organization (WHO) and the International Obesity Task Force (IOTF) to define overweight and obesity in children and adolescents by using sex- and age-specific BMI values because of the simplicity of the test method [15, 16]. The study also confirmed that WC appeared to be more valid in assessing central obesity and cardiometabolic diseases in children and adolescents compared to BMI [17, 18].. Therefore, there has been a gradual increase in the number of studies on WC and obesity in children and adolescents in recent years. However, height-induced changes in body size should also be considered when evaluating the relationship between WC and obesity. Because, children and adolescents with the same waist circumference may have different health effects depending on their height [19]. WHtR, as an important indicator for evaluating obesity and cardiometabolic risk in children and adolescents, has gradually been widely recognized by scholars in various countries [20–22].Previous studies have confirmed that WHtR is a more valid response to cardiovascular outcomes (CVD) or metabolic levels in adults than indicators such as BMI and WC [23].There have been more previous studies on WHtR and obesity and hypertension in adults and fewer studies in children and adolescents [23]. The study show that WHtR was still associated with persistent hypertension after adjusting for age, sex, and BMI z-score in children and adolescents (*p* = 0.026), suggesting a strong association between WHtR and hypertension [24].

CRF, as a core element of physical health in children and adolescents, is important for healthy development. In previous studies, more studies have focused only on the relationship between BMI and CRF, and between WC and CRF, while studies addressing WHtR and its CRF are less common. However, past studies have focused on the relationship between WHtR and cardiovascular disease in children and adolescents, and no association studies between WHtR and CRF have been found [25]. In view of the important role and significance of WHtR and CRF in relation to the health of children and adolescents. In this study, we investigated the association between WHtR and VO _2max_ in 37,081 children and adolescents aged 7–18 years in China, and to understand the association that exists between the two will provide an important reference and basis for promoting the improvement of CRF in children and adolescents.

## Methods

### Participants

This study was a cross-sectional study. The following stages were used to select participants for this study. In the first step, Heilongjiang in the north of China, Hubei in the center, Guangxi in the south, Qinghai in the west, and Jiangsu in the east were selected as the test regions for this study according to the geographic distribution of China. In the second step, one primary school, one junior high school and one senior high school were randomly selected as the test schools for this study in both urban and rural areas of each region. In the third step, in each school, all children and adolescents who met the inclusion conditions of this study were tested as test subjects. The specific conditions for the inclusion of participants in this study were: Schoolchildren and adolescents of both sexes, aged 7 to 18, born and residing in Mainland China; Selection of children and adolescents with normal intelligence and without severe physical disabilities, with the assistance of their classroom teachers; and parents and students themselves signed a written informed consent form and agreed to be tested in this study. A total of 39,741 children and adolescents aged 7–18 years from 30 schools in five regions were included in this study. After excluding 2660 invalid questionnaires after the test, 37,081 valid data were finally returned (19,125 boys, 51.6%), with a valid data recovery rate of 93.31%. Invalid questionnaires included a response rate of less than 80 per cent and missing important demographic information. The five regional samples are representative of Chinese children and adolescents. The number of boys and girls in each age group is shown in Table 1.

**Table 1:** Sample size distribution by age and sex in Chinese children and adolescents

Age(years)	Boys	Girls	Total
7 yr	2076(53.3)	1818(46.7)	3894
8 yr	1361(52.1)	1251(47.9)	2612
9 yr	1593(50.0)	1592(50.0)	3185
10 yr	1676(53.3)	1466(46.7)	3142
11 yr	1675(53.1)	1477(46.9)	3152
12 yr	1563(53.3)	1372(46.7)	2935
13 yr	1506(52.5)	1360(47.5)	2866
14 yr	1542(52.5)	1397(47.5)	2939
15 yr	1713(51.6)	1605(48.4)	3318
16 yr	1740(50.0)	1737(50.0)	3477
17 yr	1479(49.1)	1534(50.9)	3013
18 yr	1201(47.1)	1347(52.9)	2548
7–18 yr	19,125(51.6)	17,956(48.4)	37,081

Note: number (percentage) for categorical

**Fig. 1:**
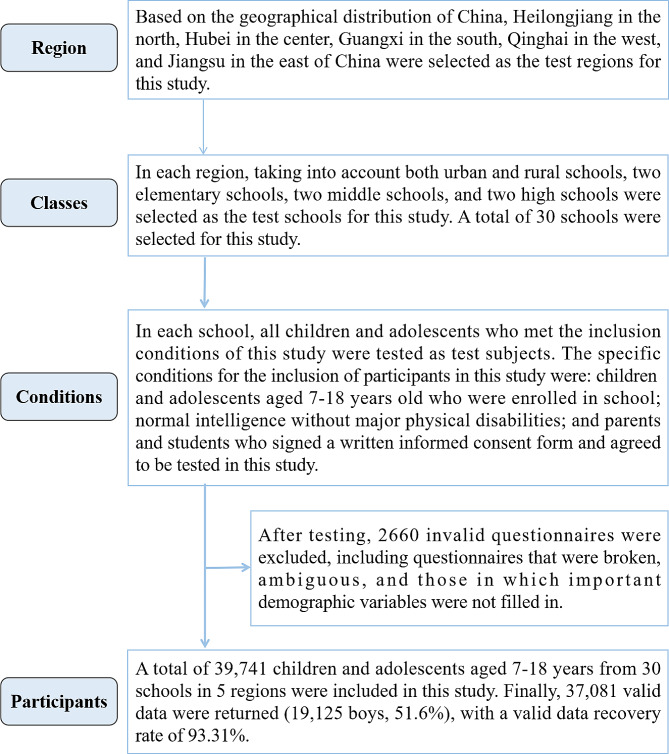
Flow chart of sampling sample size of Chinese children and adolescents

The specific sampling process for participants in this study is shown in Fig. 1.

Before the test, the staff explained the purpose, requirements, test risks and significance of the test in this study to the participants’ schools, parents and students themselves. The test card was coded for the test, and the privacy of the participants was strictly protected. This study was approved by the Ethics Committee of Guangxi Normal University (202,244,512).

### Participants’ basic information

Basic information about the participants was surveyed, including region, school, grade, class, urban/rural, birth date, and sex [26]. The age of the participants was calculated based on the birth year and test date filled in by the participants. The calculation of age in this study was based on the weekly age, i.e., the last birthday, and the test age was for children and adolescents aged 7–18 years, e.g., 18 years old was 18.0-18.9 years old.

### Height and weight

In addition, the participants’ height and weight were investigated. The height and weight measurement were conducted according to the measurement instruments and measurement methods required by the China National Student Physical Fitness and Health Research [26]. The instruments were calibrated before each day’s test to ensure the accuracy of the measurement. The measurement was conducted in the morning of each day to minimize the deviation of height in the morning and evening. The method for height was as follows: the participant stood on the test device with his/her shoes off, arms hanging down naturally, and eyes looking straight ahead in order to conduct the test accurately. For weight, the participant removes his/her shoes and wears light clothing, empties his/her bowels before the test, and stands with his/her feet on the scale in a stable position to read the weight result. The height test results are accurate to 0.1 centimeter. Weight test results are accurate to 0.1 kg [26]. The test results are filled in on a test card by the testing staff.

### Waist circumference

Waist circumference was tested according to the testing instruments and testing methods required by the China National Student Physical Fitness and Health Research [26]. The specific method was that participants wore light clothing, stood upright with their feet naturally separated, and used a soft ruler placed at the midpoint of the line between the upper edge of the hip bone and the lower edge of the twelfth rib in the right mid-axillary line, circling the abdomen in the horizontal direction for a week, close to but not pressing the skin, and measuring the length of the waist circumference at the end of the normal expiration. The test results were accurate to 0.1 centimeter [26].

The test results are filled in on a test card by the testing staff.

### Maximum oxygen uptake (VO_2max_)

Maximum oxygen uptake was tested using an indirect test method. In this study, 20-m SRT which is the most commonly used test internationally, was used to estimate the maximal oxygen uptake of the participants [27–31]. The 20-m SRT consists of two horizontal lines drawn 20 m apart on a flat surface. After warming up, the participants stood on one of the lines and ran back and forth at a pace of 1 level/min, from slow to fast, according to the rhythm of music. The tests were conducted at the school’s track and field, in groups of about 10–15 people. The initial speed was 8.0 km/h, the second was 9.0 km/h, and the speed increased by 0.5 km/h for each additional step. If the participant was unable to maintain the speed set by the music and stopped running, or if the participant was unable to reach the end line before the music started, the test was terminated, and the total number of times (laps) of the 20-m SRT was counted as the total score. Estimated peak oxygen uptake (VO_2max_,mL.kg ^− 1^ min ^− 1^ ) was calculated using an equation developed by Leger et al. based on the number of laps [32]. This is a widely used and internationally recognized method for estimating VO _2max_:

$$\begin{array}{l}{\rm{V}}{{\rm{O}}_{{\rm{2max}}}}\left( {{\rm{mL}} \cdot {\rm{k}}{{\rm{g}}^{{\rm{ - 1}}}} \cdot {\rm{mi}}{{\rm{n}}^{{\rm{ - 1}}}}} \right)\,{\rm{ = 31}}{\rm{.025 + 3}}{\rm{.238 \times S}}\\- {\rm{3}}{\rm{.248 \times Age + 0}}{\rm{.1536 \times S \times Age}}\end{array}$$

S: the running speed at the last completed stage (km.h ^− 1^ ); Age: age at the last birthday.

### Quality control

The staff involved in this study were trained and assessed prior to the test. Participants took part in the assessment only after signing an informed consent form. The test staff filled in the test results on the participants’ test cards, and the participants were asked not to alter them privately. The testing staff calibrated the testing instruments on a daily basis to guarantee the accuracy of the test results. In order to minimize the errors existing in the test, the test was conducted in the morning of each day [26].

### Statistical analysis

Continuous variables in this study were expressed as mean and standard deviation (M ± SD). The distribution of the number of people was expressed as a percentage (%). Cross-sex comparisons of continuous variables were assessed using t-tests. When analysing the data, the normality of the distribution of the variables was checked for compliance with the distribution requirements.

In this study, children and adolescents of different ages and genders were categorised by their WHtR percentile. They were classified according to the 20th, 40th, 60th, and 80th percentile WHtR cut-off value criteria as WHtR < 20th (A), 20th ≤ WHtR < 40th (B), 40th ≤ WHtR < 60th (C), 60th ≤ WHtR < 80th (D), and WHtR ≥ 80^th^ (E). Comparisons of VO_2max_ between WHtR groups were performed by one-way ANOVA. Comparison of VO_2max_ between different WHtR groups was performed using effect size between different groups. Effect was defined as small (0.20), medium(0.50), Large(0.80) [33].

Curvilinear regression analyses were performed with VO_2max_ as the dependent variable, WHtR as the independent variable, and age as a covariate. Stratified analyses were performed according to sex and age groups (7–9 year, 10–12 year, 13–15 year, 16–18 year). The relationship curve between WHtR and VO_2max_ was also plotted based on the quadratic equation. In addition, the *R*^2^ values of the equations were presented separately.

All statistics were performed and analyzed using SPSS 25.0 (IBM, Armonk, NY, USA) software and GraphPad Prism 8, with the significance level set at *p* = 0.05.

## Results

A total of 37,081 children and adolescents aged 7–18 years from 30 schools in five regions of China, including eastern, western, southern, northern and central China, were included in this study. Among them, 19,125 (51.6%) were boys and 17,956 (48.4%) were girls. The mean age of the participants was (12.38 ± 3.47) years. The mean WHtR of children and adolescents aged 7–18 years in this study was (0.42 ± 0.06) and VO_2max_ was (42.96 ± 5.40 ) mL.kg ^− 1^ min ^− 1^. Boys had higher height, weight, waist circumference, WHtR, 20-mSRT, and VO_2max_ than girls, and all differences were statistically significant (all *p* values < 0.001). Table 2.

**Table 2:** Comparison of basic status, WHtR and CRF of children and adolescents of different sexes in China (M ± SD)

Variable	Boys	Girls	Total	*t*-Value	*P*-Value
Age(years)	12.30 ± 3.44	12.47 ± 3.49	12.38 ± 3.47	4.758	<0.001
Height(cm)	155.23 ± 18.63	150.71 ± 14.73	153.04 ± 17.01	25.853	<0.001
Weight(kg)	47.69 ± 16.71	42.80 ± 12.64	45.32 ± 15.08	31.651	<0.001
Waist circumference(cm)	66.71 ± 12.33	62.73 ± 9.73	64.78 ± 11.32	34.284	<0.001
WHtR	0.43 ± 0.07	0.42 ± 0.06	0.42 ± 0.06	21.281	<0.001
20-mSRT(laps)	34.23 ± 19.60	26.39 ± 12.04	30.44 ± 16.84	46.064	<0.001
VO_2max_(mL.kg ^− 1^ min ^− 1^)	44.20 ± 4.97	41.64 ± 5.52	42.96 ± 5.40	46.999	<0.001

Note: Descriptive statistics are presented as mean (standard deviation) and number (percentage) for continuous and categorical. variables, respectively

Abbreviations: WHtR, Waist-to-height ratio; 20-mSRT, 20-m shuttle run test

The percentile division of WHtR was performed, and VO_2max_ was compared between groups by dividing them equally into quintiles using 20th, 40th, 60th, and 80th as cut-off points. The results showed that, overall, the differences in VO_2max_ between the different WHtR groups were statistically significant when compared to each other in the age groups of 7–9 year, 10–12 year, 13–15 year, and 16–18 year (*F*-value 30.06, 55.82, 8.69, and 9.27, respectively; *P* < 0.001). In the 7–9 year, 10–12 year, and 16–18 year age groups, VO_2max_ was highest in the 40th ≤ WHtR < 60th Percentile group, with (47.52 ± 2.40) mL.kg ^− 1^ min ^− 1^, (44.53 ± 3.26) mL.kg ^− 1^ min ^− 1^, and (38.38 ± 5.99) mL. ^−1^ min ^− 1^; in the 13–15 year age group, VO_2max_ was highest in the 20th ≤ WHtR < 40th Percentile group, with (42.50 ± 4.84) mL.kg ^− 1^ min ^− 1^, respectively. Comparison between different WHtR groups showed the largest effect value of 0.40 for C/E comparison in 10–12 year age group followed by 0.39 for B/E group, both with Small effect. In boys, in 7–9 yrs, 10–12 yrs, 13–15 yrs, 16–18 yrs age groups, the differences in VO_2max_ of different groups of WHtR were all statistically significant when compared to each other (*P* value < 0.001 in all cases); In girls, in the age groups of 7–9 yrs, 10–12 yrs, 16–18 yrs, the differences in VO_2max_ of different groups of WHtR were also statistically significant when compared with each other (all *P* -values < 0.001).The specific values are shown in Table 3.

**Table 3:** CRF values of Chinese children and adolescents by sex, age group and WHtR quintile

Age (yrs)	WHtR<20th Percentile(A)		20th ≤ WHtR<40th Percentile(B)		40th ≤ WHtR<60th Percentile(C)		60th ≤ WHtR<80th Percentile(D)		WHtR ≥ 80th Percentile(E)		F-value	Cohen’s d^#^									
	*N*	Mean(SD)	*N*	Mean(SD)	*N*	Mean(SD)	*N*	Mean(SD)	*N*	Mean(SD)											
**Boys**												A/B	A/C	A/D	A/E	B/C	B/D	B/E	C/D	C/E	D/E
7–9 yrs	800	47.23 ± 2.81	633	47.68 ± 2.59	903	47.77 ± 2.47	1178	47.33 ± 2.29	1516	46.89 ± 2.24	22.88^※^	0.17	0.20^a^	0.04	0.13	0.04	0.14	0.33^a^	0.18	0.37^a^	0.19
10–12 yrs	775	44.72 ± 3.62	785	45.15 ± 3.62	797	45.21 ± 3.44	1065	44.83 ± 3.32	1492	43.46 ± 3.15	54.44^※^	0.12	0.14	0.03	0.37^a^	0.02	0.09	0.50^b^	0.11	0.53^b^	0.42^a^
13–15 yrs	1045	43.67 ± 5.18	1037	44.33 ± 4.99	961	44.34 ± 5.25	860	44.00 ± 4.95	858	42.55 ± 4.76	19.33^※^	0.13	0.13	0.07	0.23^a^	0.00	0.07	0.37^a^	0.07	0.36a	0.30^a^
16–18 yrs	731	40.50 ± 5.72	908	41.56 ± 6.10	955	41.57 ± 6.19	1005	40.64 ± 6.16	821	39.27 ± 5.19	22.21^※^	0.18	0.18	0.02	0.23^a^	0.00	0.15	0.40^a^	0.15	0.40^a^	0.24^a^
**Girls**																					
7–9 yrs	855	47.02 ± 2.62	794	47.36 ± 2.32	984	47.29 ± 2.31	1080	46.86 ± 2.18	948	46.74 ± 2.05	12.59^※^	0.14	0.11	0.07	0.12	0.03	0.22	0.28^a^	0.19	0.25^a^	0.06
10–12 yrs	871	44.15 ± 3.27	922	44.00 ± 2.91	887	43.92 ± 2.97	895	43.61 ± 2.85	740	42.91 ± 2.82	21.81^※^	0.05	0.07	0.18	0.41^a^	0.03	0.14	0.38^a^	0.11	0.35^a^	0.25^a^
13–15 yrs	1066	40.61 ± 3.98	1022	40.65 ± 3.89	964	40.37 ± 3.75	804	40.31 ± 4.15	506	39.84 ± 4.10	4.36	0.01	0.06	0.07	0.19	0.07	0.08	0.20^a^	0.02	0.13	0.11
16–18 yrs	1000	35.64 ± 3.89	1147	35.72 ± 4.15	1055	35.48 ± 3.99	892	35.12 ± 3.74	524	34.35 ± 3.76	13.28^※^	0.02	0.04	0.14	0.34^a^	0.06	0.15	0.35^a^	0.09	0.29^a^	0.21^a^
**Total**																					
7–9 yrs	1655	47.12 ± 2.71	1427	47.50 ± 2.45	1887	47.52 ± 2.40	2258	47.11 ± 2.25	2464	46.83 ± 2.17	30.06^※^	0.15	0.16	0.00	0.12	0.01	0.17	0.29^a^	0.18	0.30^a^	0.13
10–12 yrs	1646	44.42 ± 3.45	1707	44.53 ± 3.30	1684	44.53 ± 3.26	1960	44.27 ± 3.17	2232	43.28 ± 3.05	55.82^※^	0.03	0.03	0.05	0.35^a^	0.00	0.08	0.39^a^	0.08	0.40^a^	0.32^a^
13–15 yrs	2111	42.13 ± 4.86	2059	42.50 ± 4.84	1925	42.35 ± 4.98	1664	42.22 ± 4.94	1364	41.55 ± 4.71	8.69^※^	0.08	0.04	0.02	0.12	0.03	0.06	0.20^a^	0.03	0.17	0.14
16–18 yrs	1731	37.69 ± 5.32	2055	38.30 ± 5.87	2010	38.38 ± 5.99	1897	38.05 ± 5.86	1345	37.35 ± 5.26	9.27^※^	0.11	0.12	0.06	0.06	0.01	0.04	0.17	0.06	0.18	0.13

Note: ^※^*P*<0.001. ^a^ Small effect = 0.20, ^b^ Medium effect = 0.50, ^c^ Large effect = 0.80. ^#^ Effect size between different groups

Abbreviation: M (SD), mean ± standard deviation

**Fig. 2:**
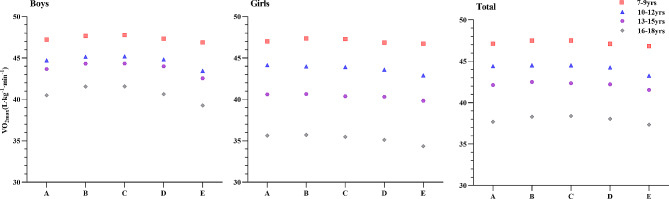
Trends of CRF in Chinese children and adolescents of different age groups and WHtR percentiles

Figure 2 shows the trend of VO_2max_ in children and adolescents of different WHtR percentiles in each age group, with respect to different sex. Overall, it can be seen that the VO_2max_ levels of Chinese children and adolescents showed a decreasing trend as the age group increased. In addition, with the increase of WHtR, VO_2max_ showed a curvilinear trend of first increasing and then decreasing.

To further understand the association between WHtR and VO_2max_, we used curvilinear regression for further analysis. We performed curvilinear regression analyses in Chinese children and adolescents using VO_2max_ as the dependent variable, WHtR as the independent variable, and age as a covariate.The results of the specific regression equations are shown in Table 4.:

**Table 4:** Curvilinear regression equations of WHtR and VO_2max_ in Chinese children and adolescents

Sex/age group	Equation	R^2^	*P*
Boys			
7–9 yrs	Y=-20.370 × ^2^ + 14.182X + 45.111	0.013	<0.01
10–12 yrs	Y=-55.863 × ^2^ + 39.758X + 38.087	0.049	<0.01
13–15 yrs	Y=-95.291 × ^2^ + 74.329X + 29.728	0.019	<0.01
16–18 yrs	Y=-133.134 × ^2^ + 106.747X + 19.843	0.02	<0.01
Girls			
7–9 yrs	Y=-16.084 × ^2^ + 10.859X + 45.394	0.006	<0.01
10–12 yrs	Y=-12.005 × ^2^ + 1.682X + 45.195	0.025	<0.01
13–15 yrs	Y=-36.634 × ^2^ + 26.081X + 35.986	0.004	<0.01
16–18 yrs	Y=-68.701 × ^2^ + 50.408X + 26.431	0.011	<0.01
Total			
7–9 yrs	Y=-16.789 × ^2^ + 11.530X + 45.408	0.008	<0.01
10–12 yrs	Y=-34.146 × ^2^ + 21.717X + 41.270	0.028	<0.01
13–15 yrs	Y=-50.482 × ^2^ + 38.968X + 34.868	0.004	<0.01
16–18 yrs	Y=-66.035 × ^2^ + 54.740X + 26.835	0.003	<0.01

Note: Y denotes VO_2max_, X denotes WHtR.

**Fig. 3:**
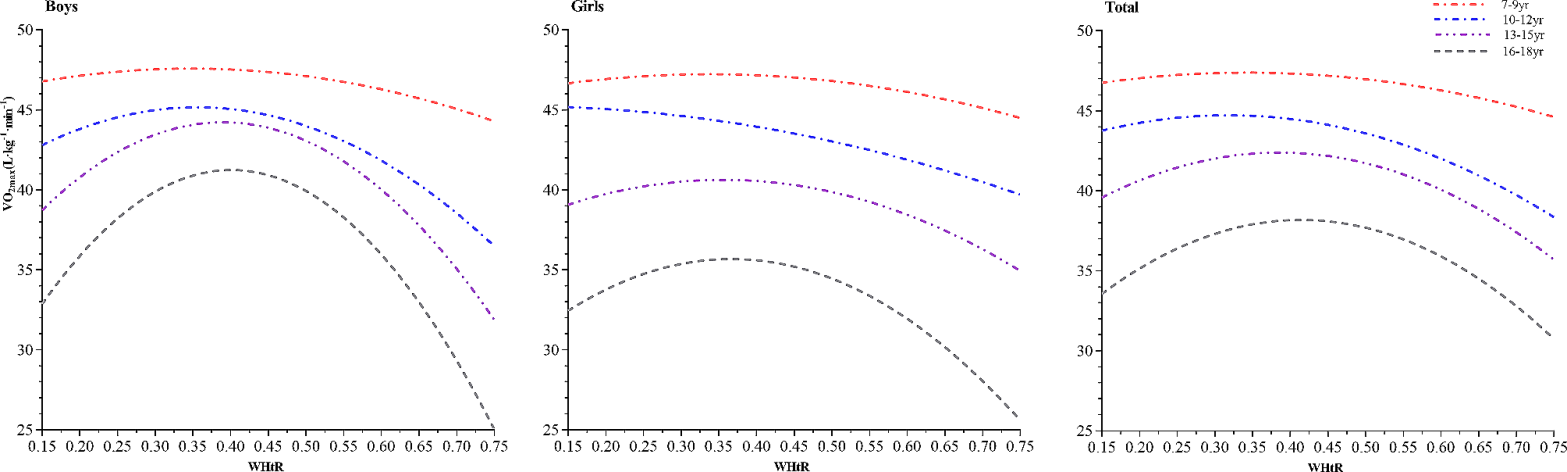
Curve relationship between WHtR and CRF in Chinese children and adolescents of different ages

We plotted the curvilinear relationship between WHtR and VO_2max_ based on the quadratic equation. Overall, it can be seen that the VO_2max_ levels of Chinese children and adolescents showed a decreasing trend with increasing age. Overall, in the 7–9 yrs age group, the highest VO_2max_ level of 47.39 mL.kg−1 min−1 was observed in children and adolescents with WHtR at 0.34. In the 10–12 yrs age group, the highest VO_2max_ level in children and adolescents was 44.72mL.kg−1 min−1 at a WHtR of 0.32. In 13–15 yrs age group, the highest VO_2max_ level in children and adolescents was 42.39mL.kg−1 min−1 at WHtR of 0.39. In the 16–18 yrs age group, the highest VO_2max_ level in children and adolescents at a WHtR of 0.41 was 38.18 mL.kg−1 min−1. In Chinese children and adolescents in the age groups of 7–9, 10–12, 13–15, and 16–18 years old, VO_2max_ levels were highest when the WHtR was 0.34, 0.32, 0.39, and 0.41, respectively. The specific trends of different sex are shown in Fig. 3.

## Discussion

The results show a gradual decrease in VO_2max_ among Chinese children and adolescents in the 7–9, 10–12, 13–15 and 16–18 age groups. However, there were some sex differences in the association between WHtR and VO_2max_. The effect of WHtR on VO_2max_ was more pronounced in boys and relatively smaller in girls. This finding is consistent with the findings of several studies [34, 35]. As the age group continues to rise its VO_2max_ shows a gradual decreasing trend its to this result is due to multiple facets. First, as age increases, body weight tends to increase, and the body’s demand for oxygen continues to rise, resulting in a gradual decrease in VO_2max_. Also, weight gain leads to lower 20-mSRT scores. In addition, as age rises, students’ academic pressure increases, resulting in prolonged screen time, increased time for static behaviors, and decreased time for physical activity, which is also an important reason for the gradual decline of VO_2max_ in children and adolescents [36]. With respect to sex, the magnitude of change in WHtR was lower in girls because they were more ambitious for a slimmer figure. On the contrary, the magnitude of change in WHtR was relatively large in boys, thus having a more pronounced effect on VO_2max_.

CRF, represented by VO_2max_, plays an important role in the health of children and adolescents and future adults. Studies have shown that good cardiorespiratory fitness is associated with a 30–40% reduction in the risk of cardiovascular disease, a 20–27% reduction in the risk of stroke, and a significant reduction in the incidence of many types of cancers such as type II diabetes, colon, breast, and lung cancers, which have a positive impact on health [37, 38]. In addition, other studies have confirmed that there is also a strong association between CRF and academic achievement in children and adolescents, and that it will affect future adult health, with a significant trajectory effect [38]. This suggests that maintaining high levels of CRF in children and adolescents has an important role and significance in promoting health. The mean VO_2max_ of the children and adolescents in this study was (42.96 ± 5.40 ) mL.kg ^− 1^ min ^− 1^, and it was significantly higher in boys than in girls. Several studies have also confirmed that VO_2max_ is significantly higher in boys than in girls, and that there is a strong association with boys’ personality factors that bring them to be more active and to spend more time on sports and exercise behaviors [2, 39, 40].

Our study is the first to analyze the association that exists between WHtR and VO_2max_ in Chinese children and adolescents. Our study showed that there were significant differences in VO_2max_ among Chinese children and adolescents with different WHtR groups compared to each other in the age groups of 7–9 year, 10–12 year, 13–15 year, and 16–18 year. Our study also observed that Chinese children and adolescents with lower or higher WHtR had lower VO_2max_. Data from previous studies show that there are fewer studies addressing the association between WHtR and VO_2max_ in children and adolescents, and the only studies that have been conducted have mainly focused on the studies of foreign scholars. In a study of 228 adults in Malaysia, a significant negative correlation was found between WHtR and VO_2max_ (*r*=−0.516) [41]. A study of 14–15 year old adolescents showed a positive correlation between WHtR and standing long jump performance, which reflects lower limb muscle strength, and a significant negative correlation with VO_2max_ [42]. We further analyzed the curvilinear relationship that exists between WHtR and VO_2max_ in Chinese children and adolescents, and our results showed that Chinese children and adolescents had the highest levels of VO_2max_ at WHtRs of 0.34, 0.32, 0.39, and 0.41 in the age groups of 7–9 year, 10–12 year, 13–15 year, and 16–18 year, respectively. Although this result we obtained cannot be used as a recommended value for WHtR or reflect the optimal threshold for CRF in Chinese children and adolescents, our study can provide necessary reference and lessons for better promotion of CRF in Chinese children and adolescents in the future.

Our study has certain strengths and limitations. Strengths: On the one hand,,this study is the first to analyze the curvilinear association between WHtR and VO_2max_ in Chinese children and adolescents, and the WHtR value derived when VO_2max_ is maximal may provide a necessary reference for effectively improving CRF in Chinese children and adolescents in the future. Second, the sample size of this study was distributed in the eastern, western, southern, northern, and central regions of China, and its results are representative. Third, although previous studies have examined the relationship between WC and VO_2max_, analyzing the relationship between WHtR and VO_2max_ will enable a more comprehensive analysis of the relationship between body composition and VO_2max_ to better facilitate the enhancement and improvement of CRF levels in children and adolescents. This study also has some limitations. First, the use of VO_2max_ to indirectly represent CRF levels in children and adolescents is not immune to some deviation from the true levels. In the future, direct testing methods reflecting VO_2max_, such as the COSMED K5 portable cardiorespiratory fitness monitor, should be used to more accurately respond to the participants’ VO_2max_ levels. Second, in the future, the optimal WHtR value suitable for Chinese children and adolescents should be recommended based on the CRF level by ROC curve analysis, and this recommended value or threshold will be able to better promote the CRF level in Chinese children and adolescents. Third, our study was a cross-sectional study, and we were unable to determine the existence of a causal relationship between WHtR and VO_2max_. Therefore, a prospective cohort study is needed in the future to further analyze the existence of a causal association between the two.

## Conclusion

A curvilinear association was found between WHtR and CRF in Chinese children and adolescents. The comparison of VO_2max_ between different WHtR groups showed significant differences in all age groups from 7 to 18 year. Both lower and higher WHtR resulted in decreased VO_2max_ in children and adolescents. A reasonable WHtR should be maintained in the future to better promote CRF in Chinese children and adolescents.

## Data Availability

No datasets were generated or analysed during the current study.
